# Unraveling the missing heritability of amyotrophic lateral sclerosis: Should we focus more on copy number variations?

**DOI:** 10.4103/NRR.NRR-D-24-01604

**Published:** 2025-04-29

**Authors:** Maria Guarnaccia, Valentina La Cognata, Giulia Gentile, Giovanna Morello, Sebastiano Cavallaro

**Affiliations:** Institute for Biomedical Research and Innovation, National Research Council, Catania, Italy

Amyotrophic lateral sclerosis (ALS) is a fatal neurodegenerative disorder characterized by the progressive degeneration of upper and lower motor neurons in the brainstem and spinal cord, leading to muscle weakness, paralysis, and respiratory failure (Morgan and Orrell, 2016). Despite identifying many genes associated with ALS risk and pathogenesis, a discrepancy exists between heritability estimates based on familial studies (40%–60%) (Al-Chalabi et al., 2010) and heritability estimates derived from genetic data (5%–10%) (Megat et al., 2023), such as those obtained through Genome-Wide Association Studies. This discrepancy, termed “missing heritability,” fuels ongoing debate and likely stems from ALS’s complex genetic architecture, limitations of current genetic research, unidentified genetic and epigenetic factors, environmental influences, random chance, and potential biases in family and twin study heritability estimates (Van Damme, 2018).

**Figure 1:**
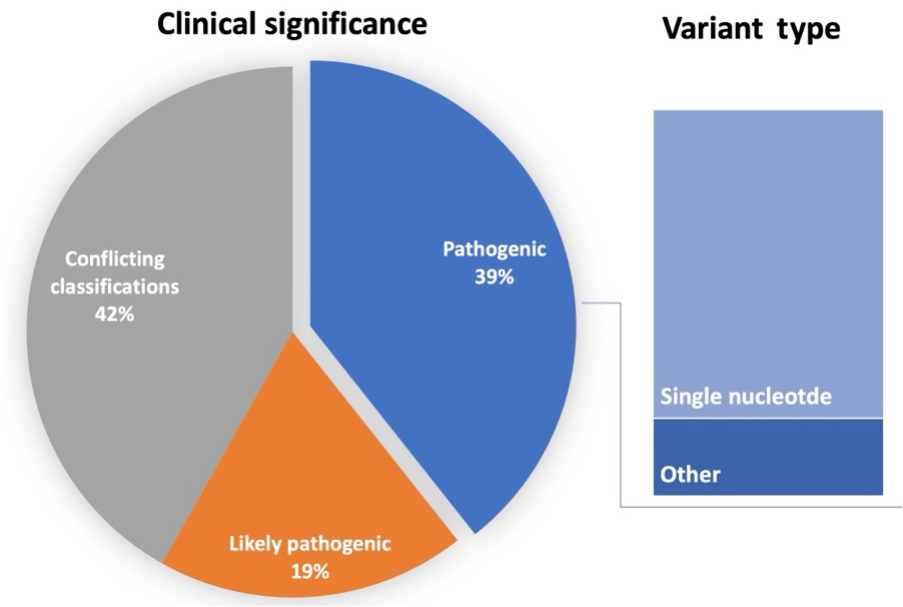
Clinical significance of all identified genetic variants in amyotrophic lateral sclerosis. Data were obtained from the ClinVar database. 39% of variants are considered pathogenic. Their predominant type is single nucleotide polymorphisms (79.6%). Other listed variants are copy number loss, deletion duplication, and insertion.

154 genes are currently implicated in ALS, exhibiting either causative or weaker associations according to ALSoD database (https://alsod.ac.uk). 681 variants are classified as pathogenic, likely pathogenic, or of uncertain pathogenicity according to ClinVar database (https://www.ncbi.nlm.nih.gov/clinvar/). Single nucleotide polymorphisms (SNPs) are the most prevalent pathogenic variant type (**Figure 1**).

However, SNP-based heritability in ALS is low (h^2^_SNP_ = 0.018) (Restuadi et al., 2022), suggesting a potentially underestimated role for other forms of genetic variation, such as copy number variations (CNVs). While CNVs are implicated in several neurodegenerative disorders, their contribution to ALS remains less explored than that of single nucleotide variants and repeat expansions (e.g., C9orf72). Several factors contribute to this underappreciation:

**Limited copy number variations studies in amyotrophic lateral sclerosis:** While CNVs in genes such as *NEK1*, *TBK1*, and *C9orf72* have been linked to ALS, comprehensive, genome-wide CNV investigations are lacking (Rizzuti et al., 2023). Some CNVs may be population-specific or rare, hindering detection in studies with limited sample sizes.

**Potential functional impact:** CNVs can alter gene dosage, disrupt coding sequences, or affect regulatory regions, impacting pathways crucial to motor neuron survival. ALS pathogenesis involves complex interactions between genetic and environmental factors, and CNVs could contribute to disease heterogeneity by modulating the effects of known risk genes (Nagy et al., 2024).

**Overlap with other neurodegenerative diseases:** CNVs implicated in Parkinson’s and Alzheimer’s diseases suggest potential shared genetic mechanisms with ALS. Overlap exists between some ALS-associated CNVs and those found in frontotemporal dementia, supporting a shared genetic basis (Firdaus and Li, 2024).

**Figure 2:**
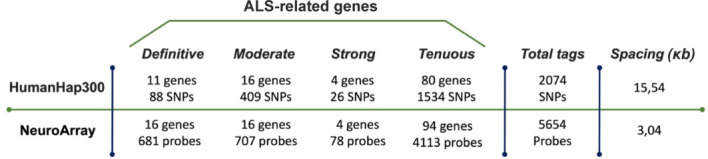
Comparison between commonly used high-density genome-wide single nucleotide polymorphisms (SNP) arrays (HumanHap300) and NeuroArray, a custom-designed exon-centric array-based comparative genomic hybridization platform. The tagSNP markers of HumanHap300 cover only a limited portion of exonic or intronic sequences in amyotrophic lateral sclerosis (ALS)-related genes, whereas NeuroArray provides high-resolution coverage of all exonic regions of ALS-related genes. Complete data are available in a paper by Guarnaccia et al. (2024).

**Limitations of current research methods:** Commonly used high-density SNP arrays often employed for CNV identification in ALS, inadequately cover genes with established pathogenic roles (**Figure 2**). Using a custom exon-centric array-based comparative genomic hybridization platform, our group has shown the presence of a high number of CNVs in ALS-related genes that could escape traditional analyses and are associated with higher onset age and disease progression rate (Guarnaccia et al., 2024).

Standard SNP-based Genome-Wide Association Studies approaches are insufficient for capturing the full spectrum of genetic variation contributing to ALS. While array-based comparative genomic hybridization remains useful, next-generation sequencing is expected to surpass it, enabling simultaneous analysis of CNVs and single nucleotide variants and streamlining the diagnostic process (Pancotti et al., 2022). Although a detailed comparison of these technologies is beyond the scope of this paper, it is worth noting that targeted next-generation sequencing of ALS-related genes is now widely employed in clinical diagnostics to identify single nucleotide variants and small indels. However, detecting CNVs from the same sequencing data remains a challenge, as different algorithms yield varying results (Smukowski et al., 2022). Several tools leverage read count data from captured regions in WGS to infer CNVs, with the most commonly used algorithms relying on circular binary segmentation and hidden Markov models. These methods depend on breakpoint detection and smoothing techniques, yet estimating coverage depth for specific genes remains difficult. Statistical biases frequently result in high false discovery rates (Zhang et al., 2024). As the standardization of these algorithms improves, guidelines should be updated to incorporate CNV detection. Integrating detailed CNV data within a systems biology framework is crucial for better understanding their role in ALS pathogenesis, ultimately aiding in patient counseling and clinical management.

In conclusion, CNVs may offer a potential explanation for ALS’s missing heritability. Determining how these genetic variants across different frequency spectrums contribute to ALS phenotypic expression is essential for advancing our understanding of the disease.
